# Pyrosequencing revealed shifts of prokaryotic communities between healthy and disease-like tissues of the Red Sea sponge *Crella cyathophora*

**DOI:** 10.7717/peerj.890

**Published:** 2015-06-11

**Authors:** Zhao-Ming Gao, Yong Wang, Ren-Mao Tian, On On Lee, Yue Him Wong, Zenon B. Batang, Abdulaziz Al-Suwailem, Feras F. Lafi, Vladimir B. Bajic, Pei-Yuan Qian

**Affiliations:** 1 Division of Life Science, The Hong Kong University of Science and Technology, Clear Water Bay, Hong Kong, PR China; 2 Sanya Institute of Deep Sea Science and Engineering, Chinese Academy of Sciences, Sanya, Hai Nan, PR China; 3 Coastal and Marine Resources Core Lab, King Abdullah University of Science and Technology (KAUST), Thuwal, Saudi Arabia; 4 Computational Bioscience Research Center (CBRC), King Abdullah University of Science and Technology (KAUST), Thuwal, Saudi Arabia

**Keywords:** Low microbial abundance, Verrucomicrobia, Sponge symbiont, Disease-like sponge

## Abstract

Sponge diseases have been widely reported, yet the causal factors and major pathogenic microbes remain elusive. In this study, two individuals of the sponge *Crella cyathophora* in total that showed similar disease-like characteristics were collected from two different locations along the Red Sea coast separated by more than 30 kilometers. The disease-like parts of the two individuals were both covered by green surfaces, and the body size was much smaller compared with adjacent healthy regions. Here, using high-throughput pyrosequencing technology, we investigated the prokaryotic communities in healthy and disease-like sponge tissues as well as adjacent seawater. Microbes in healthy tissues belonged mainly to the Proteobacteria, Cyanobacteria and Bacteroidetes, and were much more diverse at the phylum level than reported previously. Interestingly, the disease-like tissues from the two sponge individuals underwent shifts of prokaryotic communities and were both enriched with a novel clade affiliated with the phylum Verrucomicrobia, implying its intimate connection with the disease-like Red Sea sponge *C. cyathophora*. Enrichment of the phylum Verrucomicrobia was also considered to be correlated with the presence of algae assemblages forming the green surface of the disease-like sponge tissues. This finding represents an interesting case of sponge disease and is valuable for further study.

## Introduction

Sponges (phylum Porifera) are one of the oldest and most primitive metazoans (Hedges et al., 2004). They are distributed globally and are widely known to be associated with dense and diverse microbial communities of ecological and biotechnological importance (Taylor et al., 2007). Reports have shown that microbes can constitute up to 60% of the sponge tissue volume (Hentschel et al., 2003). In recent years, pyrosequencing technology has facilitated environmental microbial research and increased our knowledge concerning sponge-associated microbial diversity (Webster & Taylor, 2012). According to a recent study, up to 32 bacterial phyla and candidate phyla were documented in sponges (Schmitt et al., 2012). The sponge-species and sampling location specificity of sponge-associated microbes were also described (Lee et al., 2011; Webster et al., 2013). In addition, sponges can be grouped into high microbial abundance (HMA; previously called bacteriosponges) and low microbial abundance (LMA) sponges based on the density of microbes in the host (Hentschel et al., 2003).

An increasing number of reports have demonstrated that sponges are being threatened by disease and mortality events, most of which are accounted for increases of seawater temperature, global climate change and other independent prevailing environmental conditions (Wulff, 2006; Webster et al., 2008; Simister et al., 2012). Shifts of microbial community structures have been observed in diseased sponges (Angermeier et al., 2012; Gao et al., 2014). Microorganisms including Cyanobacteria, fungi, viruses, Alphaproteobacteria, and representatives of the genera *Bacillus* and *Pseudomonas* are considered potential pathogens that cause sponge disease (Webster, 2007). However, determining the causative agent of sponge diseases is difficult. Only one pathogen belonging to the Alphaproteobacteria and infecting the Great Barrier Reef sponge *Rhopaloeides odorabile* has been identified to date (Webster et al., 2002). Other studies have failed to establish a disease-like syndrome in healthy sponges using infection assays failed (Angermeier et al., 2011), and the roles of microbes on sponge disease remain inconclusive.

**Figure 1:**
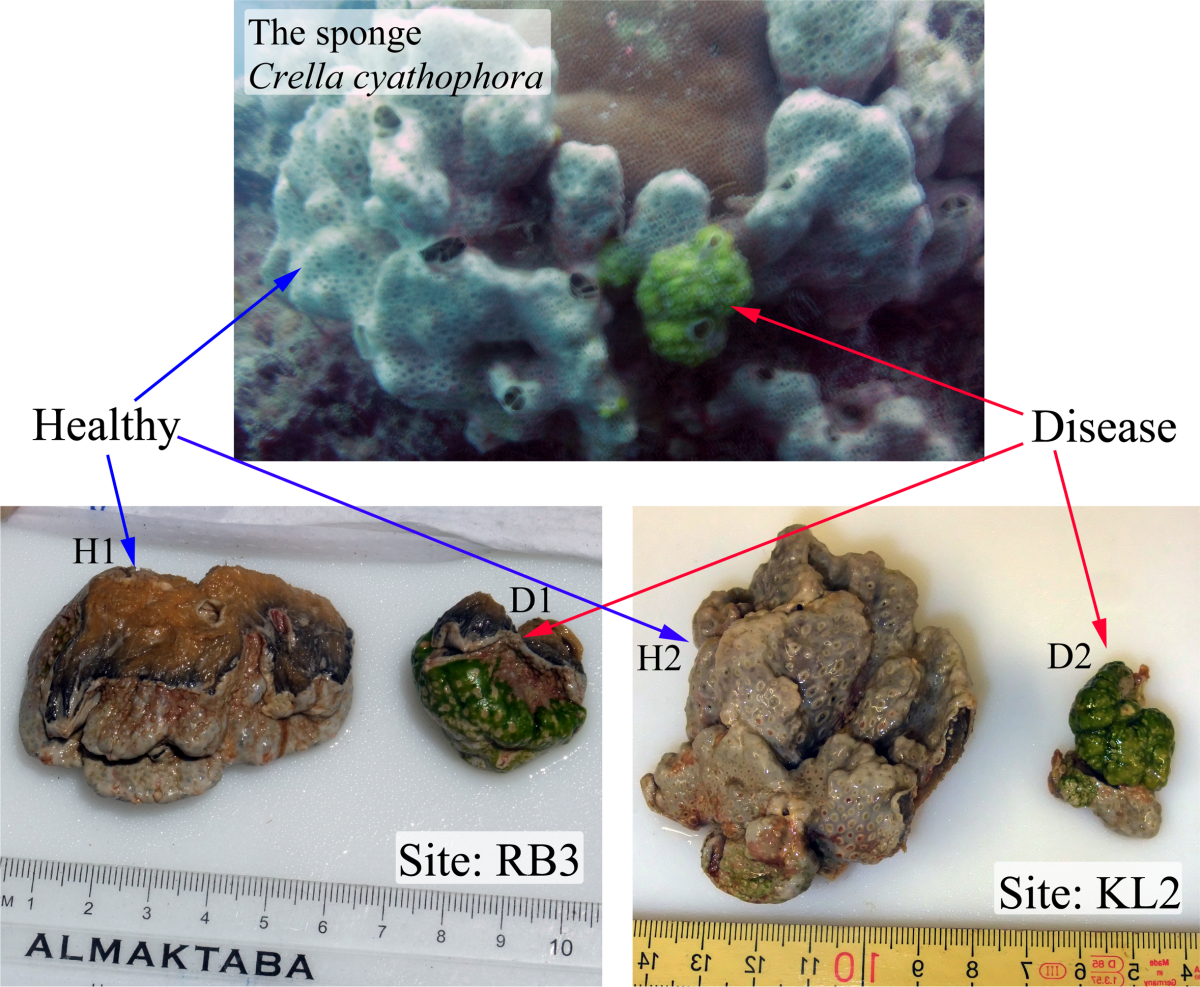
Healthy and abnormal tissues of the sponge Crella cyathophora. Sample IDs are referred to Table 1.

*Crella cyathophora* is a LMA sponge species, and its bacterial communities have been reported to be dominated by Proteobacteria (Giles et al., 2013). Here, two individuals of the LMA sponge *C*. *cyathophora* with similar disease-like characteristics were found at two different locations in Saudi Arabia along the Red Sea coast. The disease-like parts of the two individuals were both covered by green surfaces, and the body size was much smaller than adjacent healthy parts of the sponge (Fig. 1). Healthy and disease-like tissues of the two sponge individuals as well as adjacent seawater were collected and 16S rRNA genes were amplified. Using pyrosequencing of tagged 16S rRNA amplicons, shifts of prokaryotic communities in these disease-like individuals were investigated and enriched microbes in the disease-like tissues were elucidated.

## Material and methods

### Sample collection and DNA extraction

Sponge specimens and seawater were collected from two sites in Saudi Arabia along the Red Sea coast in April 2012 (Table 1 and Fig. 1). RB3 is the southern open water region off the Rabigh Bay and approximates an outfall. KL2 is located near the mouth of the Al Kharar Lagoon to the north of the Rabigh Bay and is more than 30 kilometers away from the former site. At each site, the sponge *C. cyathophora* was checked by scuba divers and disease-like individuals were targeted. The sponge species was identified with the help of Professor Rob von Soest from the Zoological Museum, University of Amsterdam. Disease-like and healthy tissues from the ectoderm of each individual were collected as well as 4 liters of seawater around each sampling site. Samples were stored in separate sterile plastic bags and transported immediately back to the laboratory. Next, sponge tissues were flushed using 0.22 µm-membrane-filtered seawater to remove loosely attached microbes and debris. The flushed tissues from each sample were separated into two parts: 10 mL for identification of sponge species was preserved in 70% of ethanol; approximately 2 × 0.5 mL for DNA extraction, which was used as two technical replicates, was dissected, cut into small pieces with a sterile razor blade, and frozen in 0.8 mL of extraction buffer (100 mM Tris–HCl, 100 mM EDTA, 100 mM Na_2_HPO_4_, 1.5 M NaCl, 1% CTAB, pH 8.0). 2 × 1 L of each seawater sample, used as two replicates, were filtered sequentially through two membranes: the first was a 1.6-µm-pore-sized glass fiber membrane (GF/A, diameter 125 mm; Whatman, Little Chalfont, UK) to remove suspended particles and eukaryotes; the second was a 0.22-µm polycarbonate membrane to capture microbial cells (polycarbonate, 47 mm in diameter; Millipore, Billerca, Massachusetts, USA). The latter polycarbonate membranes were then frozen in 0.8 mL of extraction buffer for DNA extraction. Total genomic DNA was extracted using the modified sodium dodecyl sulfate-based method described previously (Lee et al., 2011), and further purified with the Mo Bio Soil DNA Isolation Kit (Mo Bio Laboratories, Carlsbad, California, USA). The purified DNA samples were subjected to a NanoDrop ND-100 device for quantification (Thermo Fisher, Waltham, Massachusetts, USA), and then were stored at −20 °C until use.

**Table 1:** Tissue samples of the sponge *Crella cyathophora* and adjacent seawater. Samples were collected from the Rabigh Bay (RB) and Al Kharar Lagoon (KL) along the Red Sea coast.

Site	Coordinate	Depth (m)	Sample ID	Description
RB3	22°42′38″N	∼9.5	D1	Abnormal tissue (green surface) from individual 1
	38°59′47″E		H1	Healthy tissue (white surface) from individual 1
			SW3	Sea water from the site RB3
KL2	22°57′39″N	∼2.75	D2	Abnormal tissue (green surface) from individual 2
	38°49′30″E		H2	Healthy tissue (white surface) from individual 2
			SW5	Sea water from the site KL2

### PCR amplification of 16S rRNA genes and 454 pyrosequencing

The V5-V9 regions of bacterial and archaeal 16S rRNA genes were amplified by PCR using the universal primers U905F (5’-TGAAACTYAAAGGAATTG-3’) and U1492R (5’-GGTTACCTTGTTACGACTT-3’) (Leser et al., 2002; Wang & Qian, 2009). Eight-nucleotide barcodes for distinguishing sponge samples were added at the terminal of primers. A 50-µl PCR reaction volume consisted of 2.5 U of Pfu Turbo DNA polymerase (Stratagene, La Jolla, CA, USA), 1 × Pfu reaction buffer, 0.2 mM of dNTPs (TaKaRa, Dalian, China), 0.1 µM of each pair of barcoded primers and 5–10 ng of genomic DNA template. PCR was performed using a thermal cycler (Bio-Rad, Hercules, California, USA) with the following conditions: initial denaturation at 94 °C for 5 min; 30 cycles of denaturation at 94 °C for 40 s, annealing at 50 °C for 40 s and extension at 72 °C for 60 s; and a final extension at 72 °C for 5 min. PCR products were quantified using the NanoDrop device, mixed equally and subjected to the ROCHE 454 FLX Titanium platform (Roche, Basel, Switzerland) for 454 pyrosequencing. 18S rRNA genes of kinds of Eukaryotes were also amplified using the same primer pair and following the above procedure.

### Processing of sequencing data

Raw pyrosequencing data were submitted to the NCBI Sequence Read Archive under accession number SRA065210. QIIME 1.5.0 pipelines (Caporaso et al., 2010b) were used in the downstream bioinformatics analysis. First, low quality reads, including those shorter than 150 bp, those containing ambiguous bases and homopolymers of 6 bp or more and those with an average flowgram score lower than 25 in a quality windows of 50 bp, were removed. The remaining reads were assigned to respective samples according to their barcodes and subjected to Denoiser for the second round of quality control (Reeder & Knight, 2010). Singletons from Denoiser were included in the data set for the following analysis. Operational taxonomic units (OTUs) were picked at a 97% similarity from the qualified reads using UCLUST (Edgar, 2010). The most abundant read in each OTU was selected as the representative sequence. Representative reads were aligned against the SILVA 111 database with PyNAST (Caporaso et al., 2010a). The aligned sequences were searched for chimeras using ChimeraSlayer (Haas et al., 2011). Non-chimeric representatives were assigned taxonomically using the Ribosomal Database Project (RDP) classifier version 2.2 (Wang et al., 2007) against the SILVA111 database (Pruesse et al., 2007) with a confidence threshold of 0.5. Sequences that were annotated as chloroplast, mitochondria and eukaryotes were filtered out. Taxonomic abundance of reads in each sample was summarized at the phylum, class, order, family and genus levels. Species diversity, Shannon index, richness and rarefaction curves were calculated using the QIIME alpha diversity pipeline with a step size of 100 and 100 repetitions per step. Using the above mentioned primer pair, partial eukaryotic 18S rRNA genes were also successfully amplified and included in the pyrosequencing reads. They were separately summarized to reveal the relative abundance of Eukaryotes in the sponge samples.

### 16S rRNA phylogenetic tree construction

A predominant OTU assigned to the phylum Verrucomicrobia was imported into “The All-Species Living Tree” Project LTPs 115 tree (Yarza et al., 2008) and 16S rRNA sequences of the closest representative species were obtained. The OTU was further searched against the NCBI GenBank database using the BLASTn program to find closely related sequences. A neighbour-joining tree was constructed using MEGA5.1 software (Tamura et al., 2011). Multiple alignment was performed using ClustalW (Thompson, Higgins & Gibson, 1994). Distance matrices were calculated using Kimura’s two-parameter correction model (Kimura, 1980). Bootstrap values were determined with 1000 replications.

## Results and Discussion

### Diversity of sponge-associated prokaryotic communities

Two sponge individuals of the sponge *C. cyathophora* were collected, and two technical replicates of healthy and disease-like tissues were cut off from each individuals. Together with the surrounding seawater of each sampling site, a total of 12 DNA samples were obtained (Table 2). Their 16S rRNA gene amplicons were subjected to pyrosequencing. After quality filtering, 56,473 reads with an average length of 549 bp were obtained. Total number of prokaryotic OTUs in healthy sponge tissues ranged from 85 to 182, and that in disease-like tissues ranged from 76 to 142. The total number of prokaryotic OTUs was close to the number of Chao-based estimations of species richness (Table 2). Together with rarefaction curves based on observed species (OTUs, drawn using the QIIME alpha diversity pipeline at a 3% dissimilarity), the sequencing depth for all of the sponge samples was indicated to be representative, but the number of reads in seawater samples was still insufficient (Fig. S1).

**Table 2:** Pyrosequencing summary of microbial communities in sponges and seawater. OTUs, Shannon index and Chao1 were determined at 3% dissimilarity. Refer to Table 1 for sample IDs. ‘R1’ and ‘R2’ denote technical replicates 1 and 2, respectively.

Sample ID	Number of qualified reads	Total OTUs	Normalized reads (*n* = 1594)		
			OTUs	Shannon index	Chao1
D1.R1	4187	142	103	3.71	142
D1.R2	2212	81	71	2.98	105
H1.R1	3344	175	128	4.27	210
H1.R2	3449	182	134	4.71	208
D2.R1	3426	118	89	3.04	135
D2.R2	2028	76	70	3.00	92
H2.R1	3149	85	66	1.89	93
H2.R2	1662	104	102	3.22	157
SW3.R1	7552	368	191	4.95	330
SW3.R2	4707	325	210	5.41	335
SW5.R1	12598	480	186	4.99	348
SW5.R2	8159	378	178	4.75	338

The number of OTUs, Shannon index, and Chao1 estimation of species richness based on normalized reads (*n* = 1594) showed that the microbial diversity of healthy sponge samples was lower than that of seawater samples (Table 2). This finding is consistent with the low microbial diversity of the sponge *C. cyathophora* (Giles et al., 2013). The microbial diversity of disease-like sponge samples was also lower than that of seawater samples, yet no obvious divergence of microbial diversity was observed between healthy and disease-like sponge tissues.

### Taxonomic assignment of prokaryotic pyrosequencing reads at the phylum level

**Figure 2:**
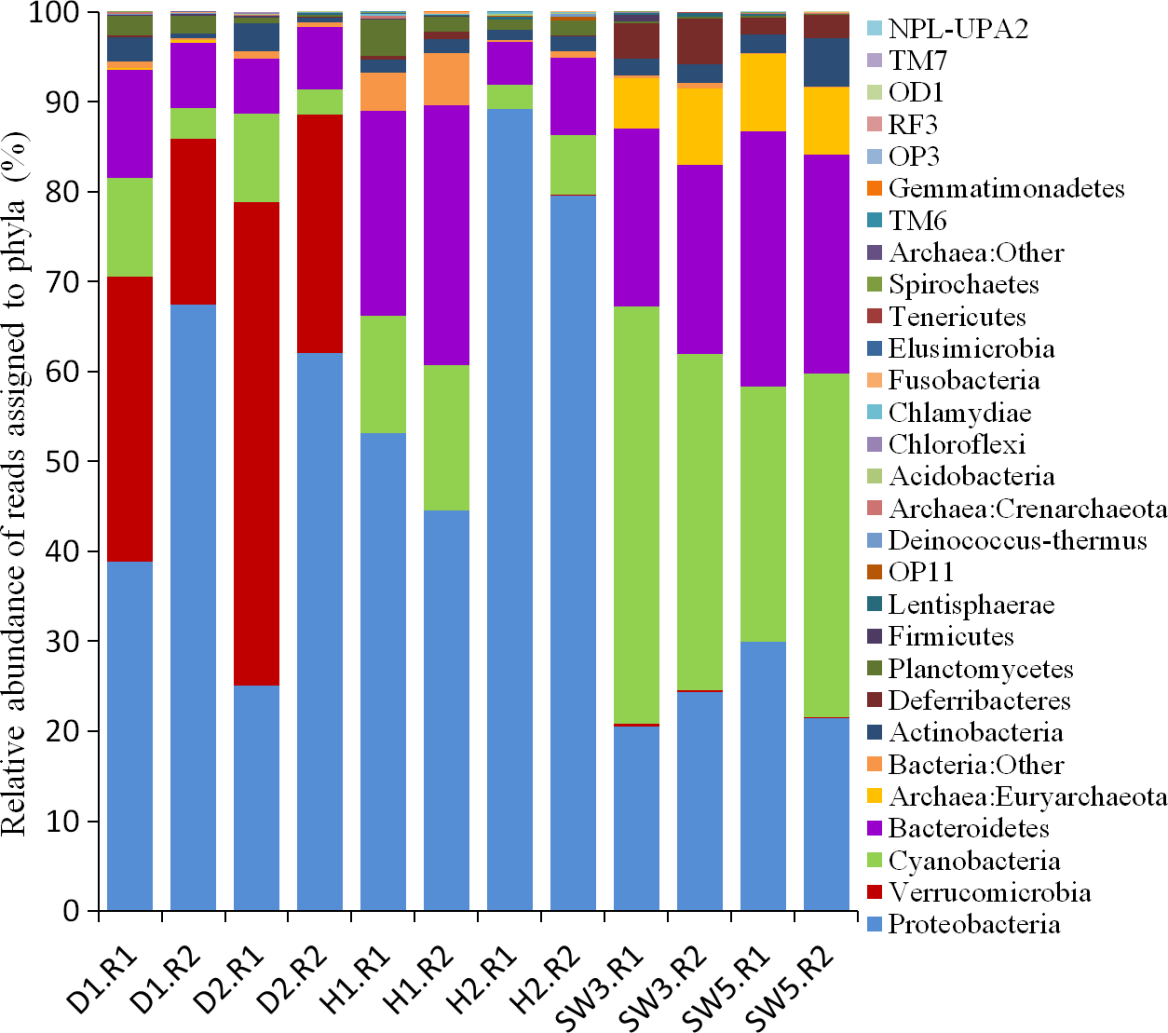
Taxonomic classification of microbial reads in sponges and seawater at the phylum level. Microbial reads were assigned taxonomically using the RDP classifier against the SILVA 111 database with a confidence threshold of 50%. Sample IDs are referred to Tables 1 and 2.

Taxonomic assignment of pyrosequencing reads at the phylum level provided insights into the shifts of prokaryotic communities in disease-like sponge tissues (Fig. 2). Healthy tissues of the sponge *C. cyathophora* were mainly composed of Proteobacteria, Cyanobacteria and Bacteroidetes. Archaea that accounted for a considerable proportion of the prokaryotic community of the surrounding seawater were rarely detected in the sponge samples, which was consistent with a previous report by Giles et al. (2013). The report also demonstrated a low phylum-level microbial diversity in the sponge *C. cyathophora* with up to only five bacterial phyla could be detected. However, using high-throughput pyrosequencing technology, we showed herein that phylum-level microbial diversity in healthy sponge tissues was higher than that reported previously, although the number of phyla that accounted for an abundance of higher than 1% of each sample was no more five (Fig. S2). In all of the disease-like sponge tissues, an additional phylum Verrucomicrobia was enriched and became predominant. The predominance of the same novel phylum in two disease-like sponge individuals collected from different locations (Table 1) should not be separated occasional events, and there should be an identical factor influencing both the two individuals.

### Taxonomic assignment of prokaryotic pyrosequencing reads at the genus level

**Figure 3:**
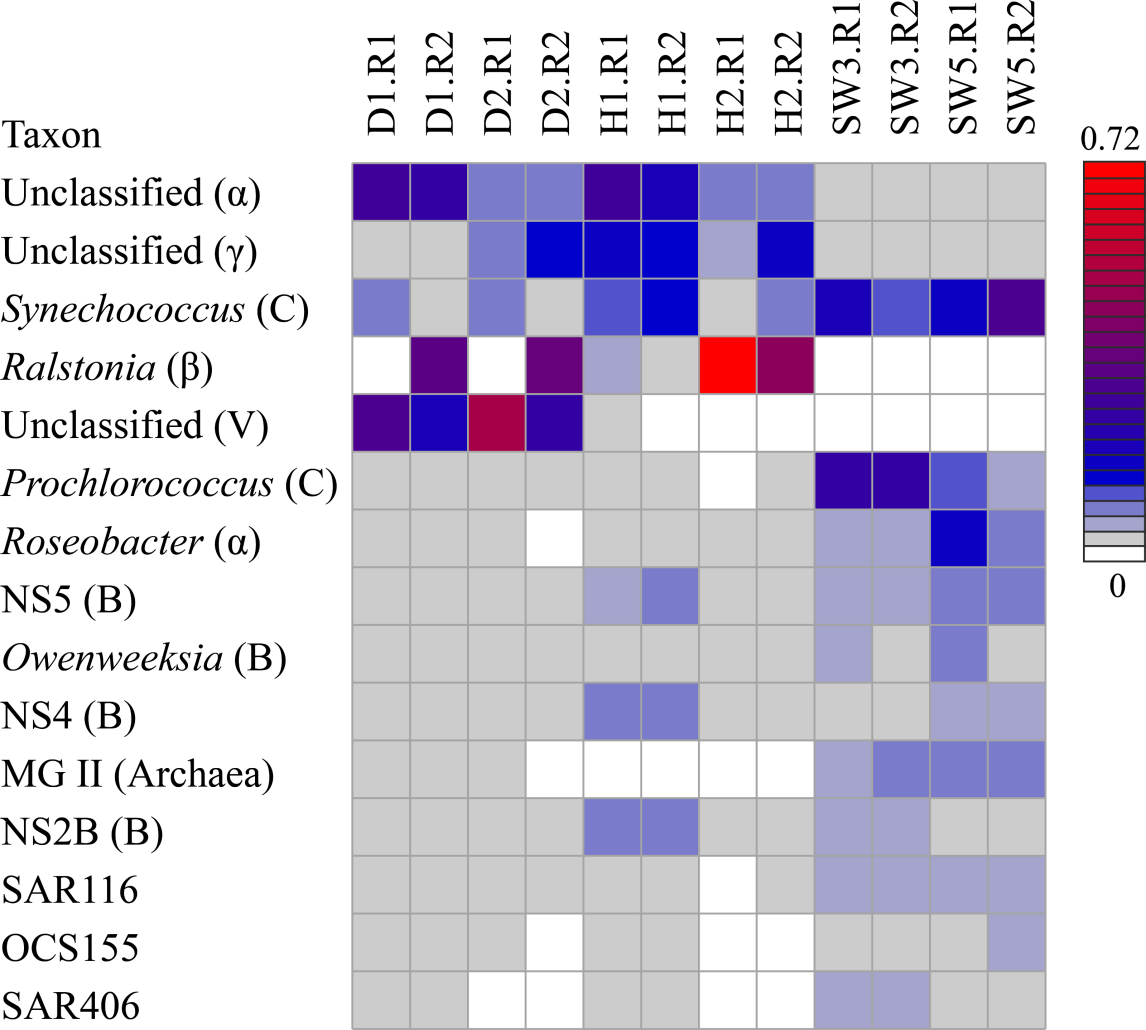
Heatmap showing the abundance of microbial reads in disease-like and healthy sponge tissues and in adjacent seawater at the genus level. Genera that showed less than 5% abundance in all the samples were filtered out. Sample IDs are referred to Tables 1 and 2. Abbreviations: *α*, Alphaproteobacteria; *β*, Betaproteobacteria; *γ*, Gammaproteobacteria; C, Cyanobacteria; B, Bacteroidetes; V, Verrucomicrobia.

Taxonomic assignment of pyrosequencing reads at the genus level was further summarized and the abundance of the genera that showed more than 5% abundance in at least one sample was depicted in a heatmap (Fig. 3). Unclassified Alphaproteobacteria and Gammaproteobacteria showed a high abundance in healthy sponge tissues as well as in several disease-like tissues, yet they were rarely found in surrounding seawater. Unclassified Alphaproteobacteria and Gammaproteobacteria also have been reported to be dominant in the sponge *C. cyathophora* according to a previous study (Giles et al., 2013), which implied that these uncultured bacteria are key sponge-associated components. LMA sponges were reported to share more microbes with surrounding seawater than HMA sponges (Moitinho-Silva et al., 2014). The genus *Synechococcus*, which was highly abundant in surrounding water, could also be found in several healthy and disease-like sponge samples. Alternatively, there was a random abundance pattern of the genus *Ralstonia* among samples. Although the genus *Ralstonia* could not be detected in adjacent seawater, it was only highly abundant in healthy tissues of sponge individual 2 but not in individual 1. It was present as a significant proportion of the disease-like samples D1.R2 and D2.R2, but was missing in the other two disease-like samples. Previous studies have reported the spatial distribution of sponge-associated prokaryotic communities, and some microbes were only found in the endosome of the sponge *Tethya aurantium* (Thiel et al., 2007). Our study focused on the ectoderm of the sponge *C. cyathophora*, but some samples have also included endosome tissues, which maybe the reason causing the large difference between disease-like technical replicates. *Ralstonia* is usually heterotrophic and rarely reported in sponge-associated microbial communities. Detection of the genus *Ralstonia* in the sponge *C. cyathophora* is curious, but it may represent a novel sponge-associated bacterium that was overlooked in other sponge-related studies.

**Figure 4:**
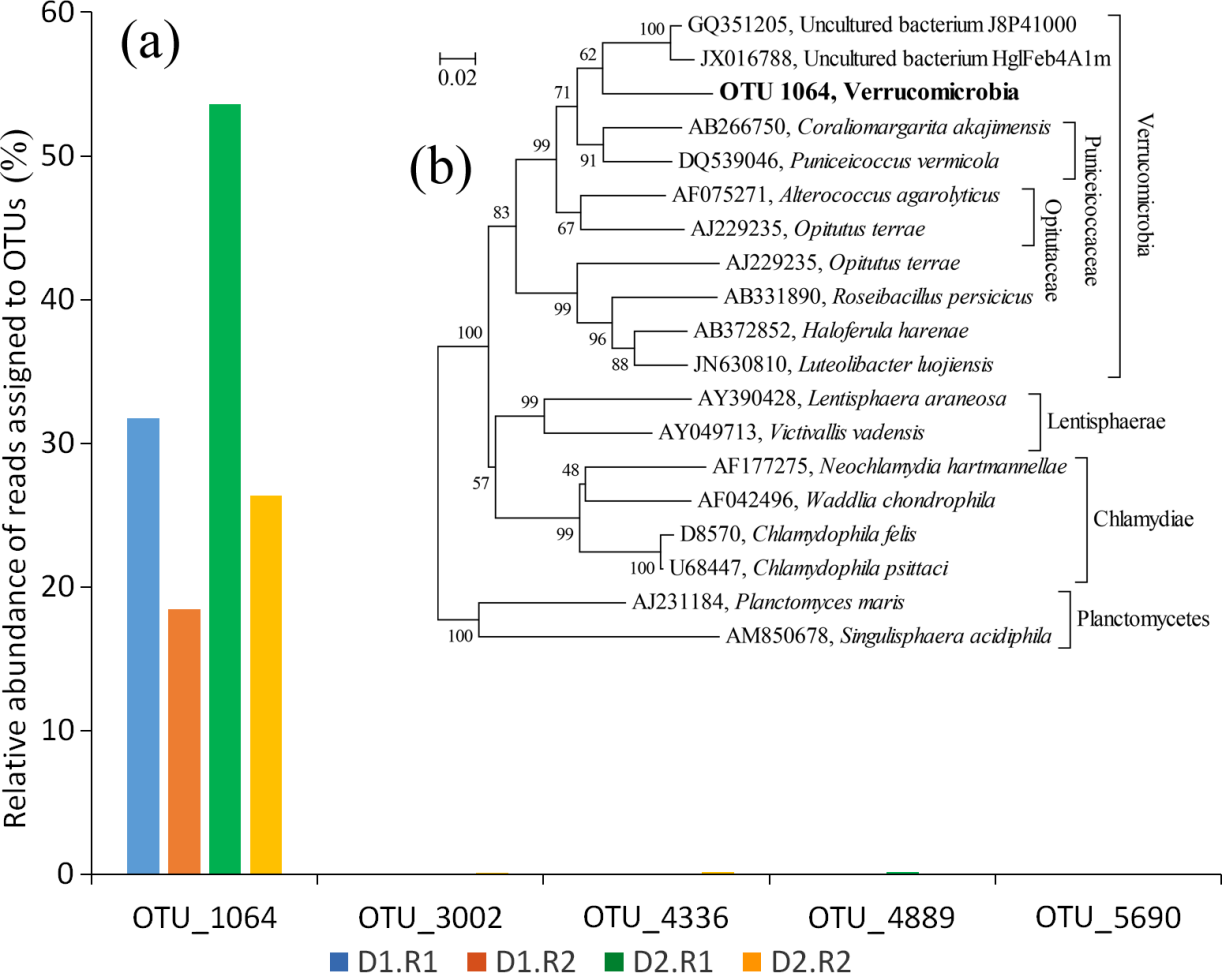
The abundance of OTUs affiliated with the phylum Verrucomicrobia and the phylogenetic relationship of OTU_1064. (A), proportions of the OTUs in disease-like tissues of the sponge *Crella* cyathophora; (B), the phylogenetic relationship of OTU_1064 with representative species in the LTPs 115 tree and uncultured clones in the NCBI GenBank NR database. The tree was constructed based on the neighbor-joining method. Values are expressed based on 1000 replications. Bar, 0.02% estimated sequences divergence.

Interestingly, the phylum Verrucomicrobia, which was specifically enriched in disease-like sponge tissues fell into a single unclassified cluster at the genus level (Fig. 3). An in-depth analysis showed that the verrucomicrobial bacteria belonged mainly to a single OTU (OTU_1064) that became dominant in the disease-like sponge tissues and occupied up to 53% of the prokaryotic communities (Fig. 4). OTU_1064 formed a deep clade in the phylum Verrucomicrobia and showed a high dissimilarity with the representative identified species. A BLASTn search further showed that OTU_1064 shared less than 90% identity with all of the sequences stored in the GenBank NR database. This finding suggested the presence of a novel verrucomicrobial clade with a function that cannot be predicted based on its close relatives. Enrichment of this novel clade implied its intimate connection with the disease-like sponge tissues and likely represents a pathogen infecting the disease-like sponge tissues. However, there is also the possibility that the potential disease attracted this novel bacterium into the sponge tissues. The two disease-like sponge individuals were both covered with a green surface, which might be assemblages of algae. There is a large possibility that this novel Verrucomicrobia clade correlated with the assumed algal assemblages. We also identified 18S rRNA gene sequences of green algae affiliated with the class Prasinophyceae (Table S1), although it still could not be sure whether these are the assumed algal assemblages.

The primer pair we used as above could also successfully amplify eukaryotic 18S rRNA genes, and the relative abundance of eukaryotic communities was also summarized at phylum, order, class and genus level. Green algae affiliated with the class Prasinophyceae dominated the eukaryotic communities of the sponge samples (Table S1), and were potentially related to the green assemblages. Yet, it still could not be sure whether these are the assumed algal assemblages because Prasinophyceae appeared both in healthy and diseased-like sponges tissues.

## Conclusions

Here, using high throughput pyrosequencing technology, we re-analyzed the prokaryotic communities of this sponge species and their shifts in disease-like tissues. Healthy tissues showed a low microbial diversity and were dominated by Proteobacteria, as reported previously (Giles et al., 2013). Disease-like tissues from two sponge individuals underwent shifts of prokaryotic communities, and both showed enrichment for a novel verrucomicrobial clade. Because there were only two individuals, the extent of the disease spreading could not be evaluated. However, the finding of disease-like sponges at two distinct locations was interesting. The occurrence of a novel verrucomicrobial clade in the disease-like sponge tissues with a small body size and covered by green surfaces certainly indicated an intimate relationship between this novel clade and the disease-like Red Sea sponge *C. cyathophora*. Collecting more biological sponge replicates that display this disease-like phenomenon from different locations of the Red Sea is required to re-assess the prevalence of this novel verrucomicrobial clade. In addition, further studies are also needed to uncover the source of the novel verrucomicrobial clade and its ascertained roles in disease-like sponges.

## Supplemental Information

10.7717/peerj.890/supp-1 Table S1 Taxonomic abundance of Eukaryotes in the sponge *Crella cyathophora* based on partial 18S rRNA gene sequences The abundance was calculated as the proportions of reads of each eukaryotic clade against all reads of Eukaryotes in each sample.

10.7717/peerj.890/supp-2 Figure S1 Rarefaction curves showed the microbial diversity in sponges and seawater The curves were drawn based on the number of OTUs at a 3% dissimilarity level. Sample IDs were referred to Tables 1 and 2.

10.7717/peerj.890/supp-3 Figure S2 Number of phyla detected in sponges and adjacent seawater The black bar indicated total number of microbial phyla in each sample, and the blank bar indicated the number of phyla of which the abundance in microbial communities is higher than 1%. Sample IDs were referred to Tables 1 and 2.

## Additional Information and Declarations

### DNA Deposition

The following information was supplied regarding the deposition of DNA sequences: Raw pyrosequencing data were submitted to the NCBI Sequence Read Archive under accession number SRA065210.
