# Supplementary material for: Pyrosequencing revealed shifts of prokaryotic communities between healthy and disease-like tissues of the Red Sea sponge Crella cyathophora
Source: PeerJ. 2015 Jun 11;3:e890. doi: 10.7717/peerj.890 (PMC4465955; doi:10.7717/peerj.890)
Supplement: Table S1 — The abundance was calculated as the proportions of reads of each eukaryotic clade against all reads of Eukaryotes in each sample. [file peerj-03-890-s001.docx]

**Table S1.** Taxonomic abundance of Eukaryotes in the sponge *Crella cyathophora* based on partial 18S rRNA gene sequences.

The abundance was calculated as the proportions of reads of each eukaryotic clade against all reads of Eukaryotes in each sample.

| Taxon | D1.R1 | D1.R2 | D2.R1 | D2.R2 | H1.R1 | H1.R2 | H2.R1 | H2.R2 |
| --- | --- | --- | --- | --- | --- | --- | --- | --- |
| Alveolata | 0.0667 | 0.0461 | 0.1863 | 0.1316 | 0.0593 | 0.1202 | 0.3416 | 0 |
| Annelida | 0 | 0 | 0 | 0 | 0.0007 | 0.0055 | 0 | 0 |
| Arthropoda | 0.0023 | 0 | 0 | 0.0789 | 0.0126 | 0.0437 | 0 | 0 |
| Chordata | 0.0056 | 0 | 0 | 0.2368 | 0 | 0 | 0 | 0 |
| Cnidaria | 0 | 0 | 0 | 0 | 0 | 0.0219 | 0 | 0 |
| Cryptophyta | 0 | 0 | 0 | 0 | 0 | 0 | 0.0082 | 0 |
| Fungi | 0 | 0 | 0 | 0 | 0 | 0.0055 | 0.0123 | 0 |
| Ichthyosporea | 0 | 0 | 0 | 0 | 0 | 0 | 0.0247 | 0 |
| Nematoda | 0 | 0 | 0.0098 | 0 | 0.0014 | 0.0055 | 0 | 0 |
| Platyhelminthes | 0 | 0 | 0 | 0 | 0 | 0.0055 | 0 | 0 |
| Rhizaria | 0 | 0.0046 | 0.1961 | 0.0263 | 0.0244 | 0.2077 | 0 | 0 |
| Rhodophyta | 0 | 0 | 0 | 0 | 0 | 0.0109 | 0 | 0 |
| Stramenopiles | 0 | 0 | 0 | 0.0526 | 0 | 0.0055 | 0 | 0 |
| Viridiplantae | 0.5028 | 0.4608 | 0.4314 | 0.3421 | 0.4826 | 0.2350 | 0.5185 | 0.8889 |
| **Class: Prasinophyceae** | **0.5017** | **0.4608** | **0.4314** | **0.3421** | **0.4784** | **0.2350** | **0.5185** | **0.8889** |
| Genus: Tetraselmis | 0.0011 | 0 | 0 | 0 | 0.0042 | 0 | 0 | 0 |
| Environmental samples | 0.4226 | 0.4885 | 0.1765 | 0.1316 | 0.4191 | 0.3333 | 0.0947 | 0.1111 |
