# Supplementary figures and images for: Pyrosequencing revealed shifts of prokaryotic communities between healthy and disease-like tissues of the Red Sea sponge Crella cyathophora
Source: PeerJ. 2015 Jun 11;3:e890. doi: 10.7717/peerj.890 (PMC4465955; doi:10.7717/peerj.890)

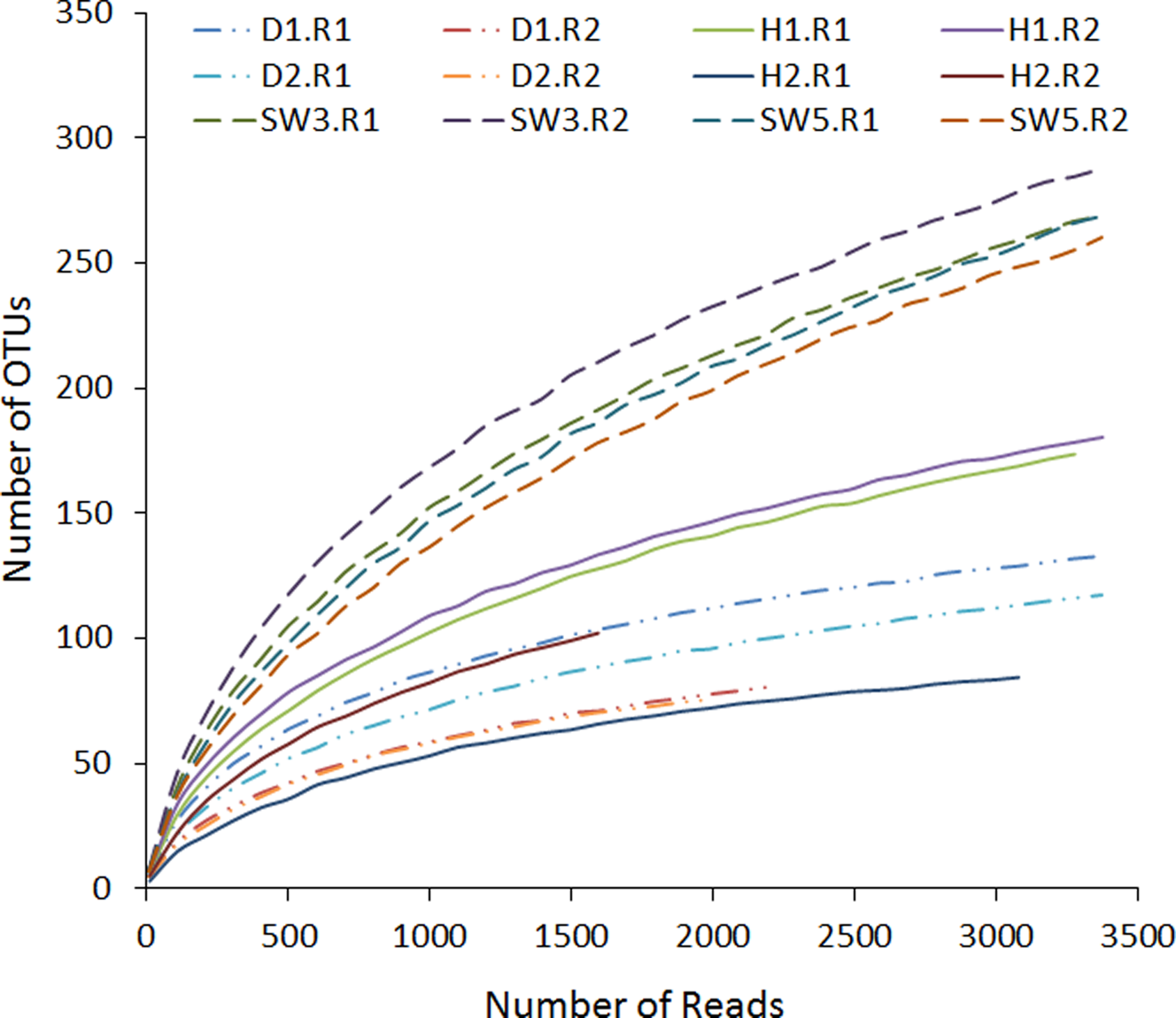

Supplement: Figure S1 — The curves were drawn based on the number of OTUs at a 3% dissimilarity level. Sample IDs were referred to Tables 1 and 2. [file peerj-03-890-s002.png]

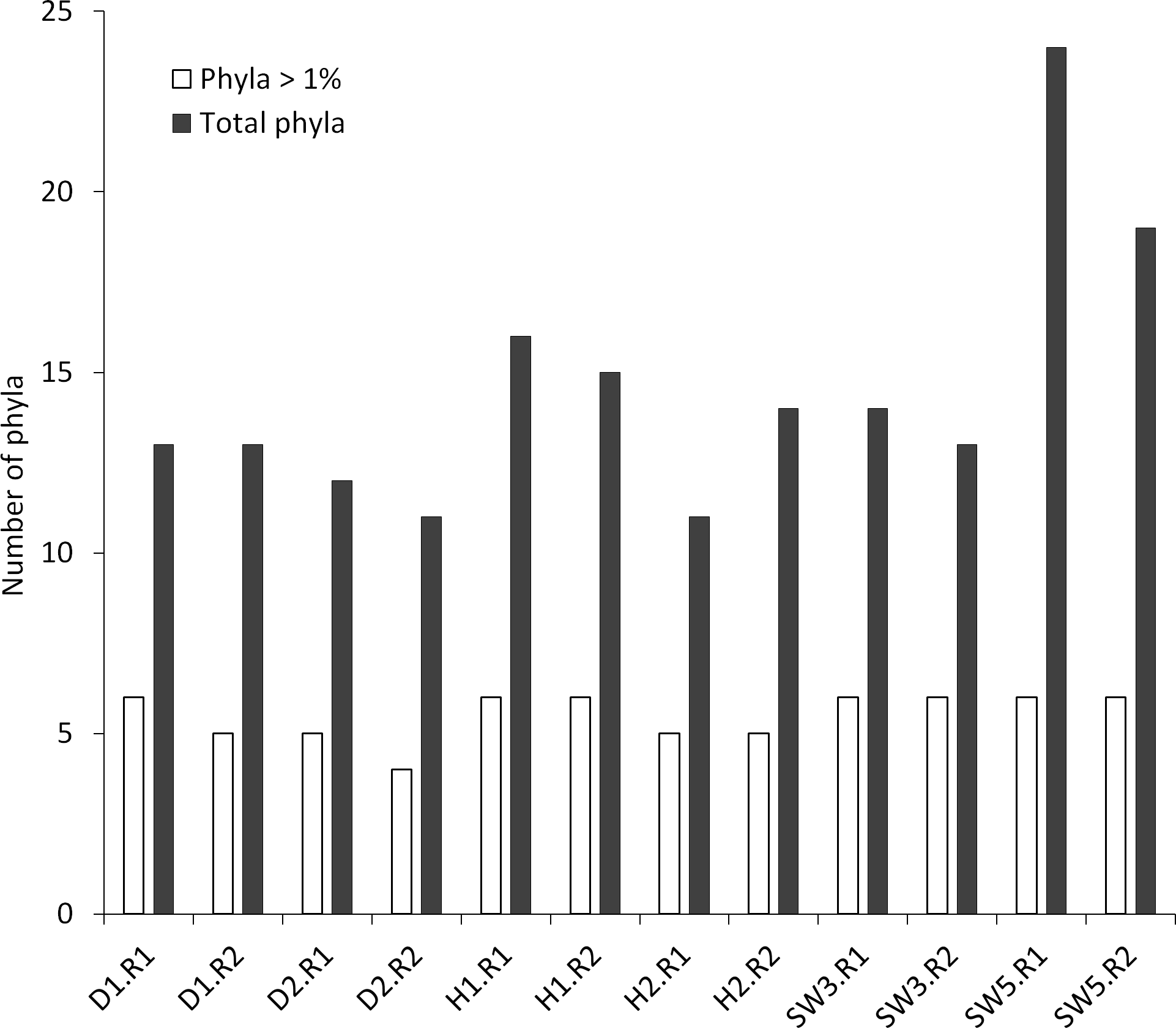

Supplement: Figure S2 — The black bar indicated total number of microbial phyla in each sample, and the blank bar indicated the number of phyla of which the abundance in microbial communities is higher than 1%. Sample IDs were referred to Tables 1 and 2. [file peerj-03-890-s003.png]
